# Multivisceral IgG4-related disease presenting as recurrent massive gastrointestinal bleeding: a case report and literature review

**DOI:** 10.1186/s12876-018-0867-y

**Published:** 2018-09-04

**Authors:** Xuexue Deng, Ronghua Fang, Jianshu Zhang, Rongqiong Li

**Affiliations:** Department of General Medicine, West China Hospital, Sichuan University, No. 37 Guoxuexiang, Wuhou District, Chengdu City, Sichuan Province China

**Keywords:** IgG4-related disease, Plasma cells, Liver, Gallbladder, Duodenal, Pancreatic, Gastrointestinal bleeding

## Abstract

**Background:**

IgG4-related disease (IgG4-RD) is a newly recognized autoimmune systemic disorder characterized by elevated levels of serum IgG4 and abundant infiltration of IgG4-positive plasmacytes in the affected organs. The liver, biliary system and pancreas are the most commonly affected organs. However, involvement of the digestive tract is very rare. To date, only a few cases of isolated gastric IgG4-RD have been reported.

**Case presentation:**

We present a case of IgG4-RD of the liver, gallbladder, pancreas and duodenum, which was clinically misinterpreted and thereafter over-treated. A 52-year-old male presented with obstructive jaundice for 3 years, melena for 5 months and hematemesis for 10 days. Three years prior, the patient had undergone biopsies of pancreatic lesions, liver lesions, cholecystectomy and choledochojejunostomy. Histopathology showed chronic inflammatory changes. Endoscopy at admission revealed a duodenal ulcer with active bleeding. Despite medical management, the patient presented with repeated gastrointestinal bleeding. Upon evaluation, serum IgG4 levels were found to be elevated. Histopathology of the duodenal ulcer biopsy and repeated examination of the gallbladder and pancreatic and liver biopsies confirmed IgG4 positive plasma cell infiltration. A definitive diagnosis of IgG4-RD was made and steroid administration was initiated. At last follow up, 11 months to-the-day after initiating steroid treatment, the patient was asymptomatic.

**Conclusions:**

Notably, IgG4-RD of multiple digestive organs is still very rare. As a systemic disease, it is characterized by the infiltration of IgG4-bearing plasma cells and raised IgG4 levels. Histopathology findings remain the diagnostic gold standard for this disorder.

## Background

IgG4-related disease (IgG4-RD) is a relatively recently recognized chronic multi-organ autoimmune disease of unknown origin with a tendency to develop lesions at multiple sites throughout the body [1]. It is known to affect various organs including the bile duct, pancreas, gallbladder, liver, lungs and salivary glands. It is characterized by elevated IgG4 levels in the serum and the affected tissues, lymphoproliferative infiltration and sclerotic fibrosis [2, 3]. Other typical features are occlusive phlebitis and rising IgG4-plasma cells levels [4, 5]. Clinically, it mimics malignancy in some patients, especially those with liver and pancreatic lesions and is often thought to necessitate surgical resection, sometimes leading to over-treatment [6, 7]. To date, only a few isolated cases of digestive IgG4-RD have been reported.

## Case presentation

A 52-year-old male from southwest China presented with obstructive jaundice for 3 years, melena for 5 months and hematemesis for 10 days. The patient had been diagnosed with type 2 diabetes mellitus for 4 years, which was treated with metformin. The patient was a chronic tobacco (10 cigarettes per day) and alcohol consumer over the previous 20 years but had quit smoking and drinking for the past 3 years. Three years prior to admission to our hospital, he developed obstructive jaundice, which was investigated with abdominal computed tomography (CT) and revealed multiple hypodense lesions in the liver and pancreatic head mass. He underwent cholecystectomy, choledochojejunostomy and biopsies from the liver and pancreatic lesions. Histopathology revealed chronic cholecystitis with lymphocytic, plasmacytic and eosinophilic infiltration of the gallbladder, liver and pancreatic parenchyma.

The physical examination was unremarkable (height − 165 cm, weight – 50 kg). Laboratory tests revealed hemoglobin levels of 75 g/L, a platelet count of 80 × 10^9^/L, 30.0 g/L albumin levels, and serum potassium levels of 3.20 mmol/L (Table 1). The fecal occult blood was positive.

**Table 1:** Comparison of IgG4 parameters and routine blood and markers for liver function before and after treatment

Parameter	Admission day	Confirmed day	After treatment 8 days	Follow up 1 month	Follow up 3 months	Follow up 6 months	Follow up 9 months
IgG4 (g/L)	–	29.20	18.40	9.86	4.74	1.87	1.70
IgG (g/L)	–	24.5	19.4	–	–	8.80	–
CRP (mg/ L)	5.46	–	–	–	2.62	1.32	–
TBIL (μmol/L)	12.1	11.7	56.4	23.4	14.1	7.7	–
DBIL (umol/L)	6.5	5.4	44.5	16.4	6.1	3.1	–
ALT (IU/L)	41	13	315	134	44	29	–
AST (IU/L)	40	24	167	57	28	26	–
ALB (g/L)	30	36.4	34.8	37.1	35.2	42.1	–
PLT(*10^9^/L)	80	95	88	106	81	110	140
HGB (g/L)	75	61	98	71	122	114	123

*IgG4* Immunoglobulin G4 subtype, *IgG* Immunoglobulin G, *CRP* C-reactive protein, *TBIL* total bilirubin, *DBIL* direct bilirubin, *ALT* alanine aminotransferase, *AST* aspartate aminotransferase, *ALB* albumin, *PLT* platelet, *HGB* hemoglobin

**Fig. 1:**
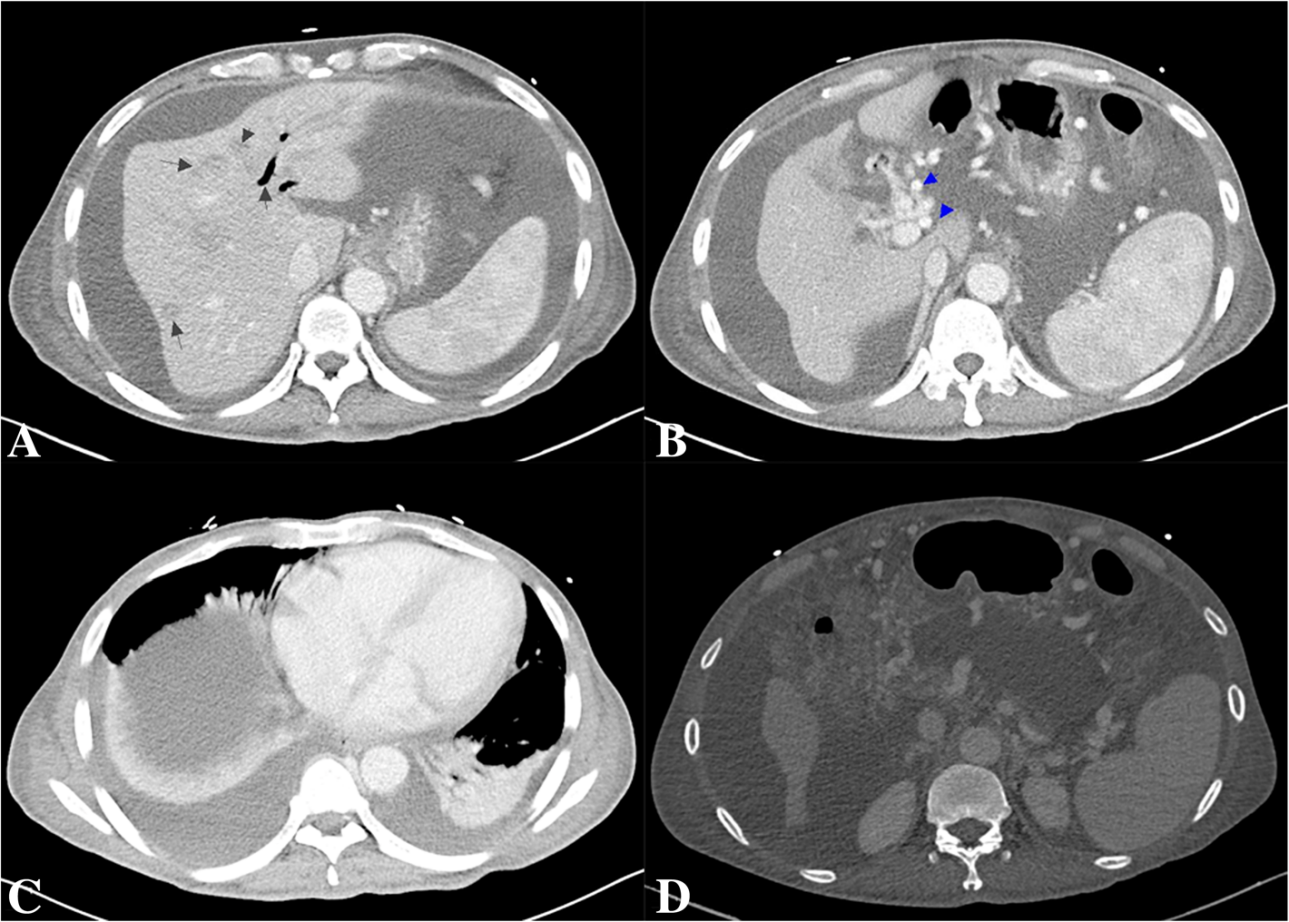
Abdominal computed tomography. Images of the liver showed numerous hypodense nodules with mild contrast enhancement, ascites and intrahepatic bile duct dilatation (**a**). There were multiple enlarged lymph nodes in the hepatoduodenal ligament around the abdominal aorta (**b**). Mesenteric lymph nodes with bilateral pleural effusion (**c**). Multiple nodular dense shadows with dilatation of the main pancreatic duct in the tail region (**d**)

**Fig. 2:**
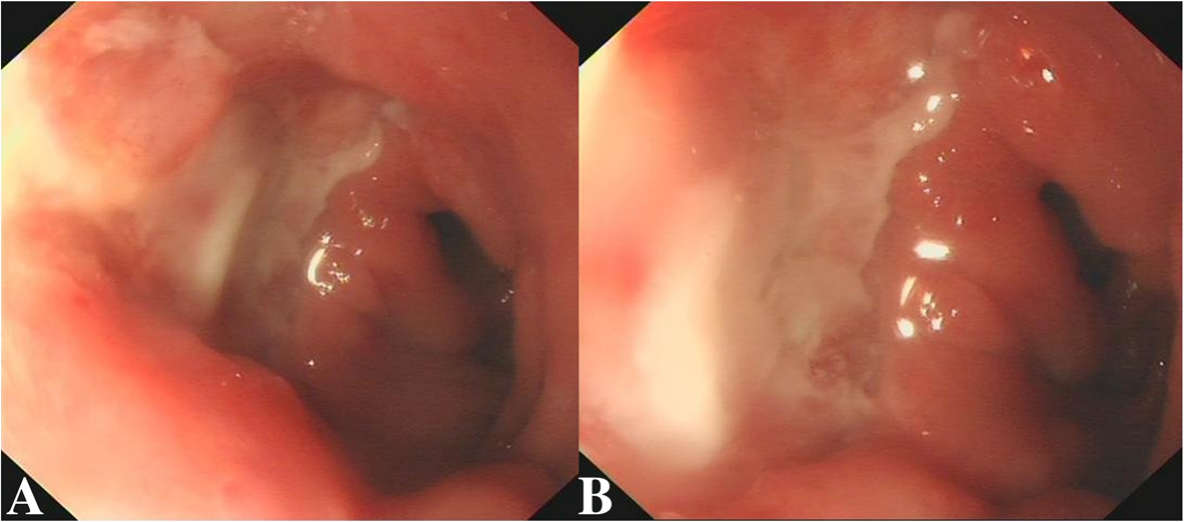
Gastroscopy findings. An ulcer (1.5 cm × 1.2 cm) in the anterior wall of the duodenal bulb and a lot of white mosses in the fundus of stomach (**a**, **b**)

On the day of admission, the patient developed repeated painless hematemesis of about 2100 mL, which was associated with hypotension and tachycardia. He was resuscitated with intravenous, blood and norepinephrine infusions. Laboratory tests revealed hemoglobin levels of 47.5 g/L, platelet count levels of 38 × 10^9^ L and serum albumin 25.8 g /L. The day after admission, the patient again developed hematemesis of about 500 mL, for which he was treated with somatostatin, terlipressin and pantoprazole infusion. Sengstaken-Blakemore tube were placed to hemostasis by compression and tube feeding hemostatic medicine. After 4 days of medical intervention, the patient’s gastrointestinal bleeding gradually stopped. Esophagogastroduodenoscopy revealed a duodenal ulcer (A1 stage) with active bleeding, gastric mucosal erosions and esophageal varices (mild). Contrast enhanced computed tomography of the abdomen with three-dimensional reconstruction exposed multiple nodular dense shadows with dilatation of the main pancreatic duct in the tail region (Fig. 1d). Images of the liver showed numerous hypodense nodules with mild contrast enhancement, ascites and intrahepatic bile duct dilatation (Fig. 1a). Additionally, there were multiple enlarged lymph nodes in the hepatoduodenal ligament around the abdominal aorta (Fig. 1b) and mesenteric lymph nodes with bilateral pleural effusion (Fig. 1c). In view of the liver disease with portal hypertension and esophagogastric varices, a transjugular intrahepatic portosystemic shunt (TIPS) procedure was performed under local anesthesia and regional portal hypertension, splenic vein and portal vein obstruction were detected. Subsequently, the patient re-bleed both 14 days and 20 days after admission, about 600 mL and 400 mL, respectively. The patient was treated with somatostatin, pantoprazole infusion and blood transfusion. Gastroscopy identified an ulcer (1.5 cm × 1.2 cm) in the anterior wall of the duodenal bulb that was not actively bleeding (Fig. 2a, Fig. 2b). Pathological examination of the ulcer biopsy verified a moderate degree of chronic mucosal inflammation.

**Fig. 3:**
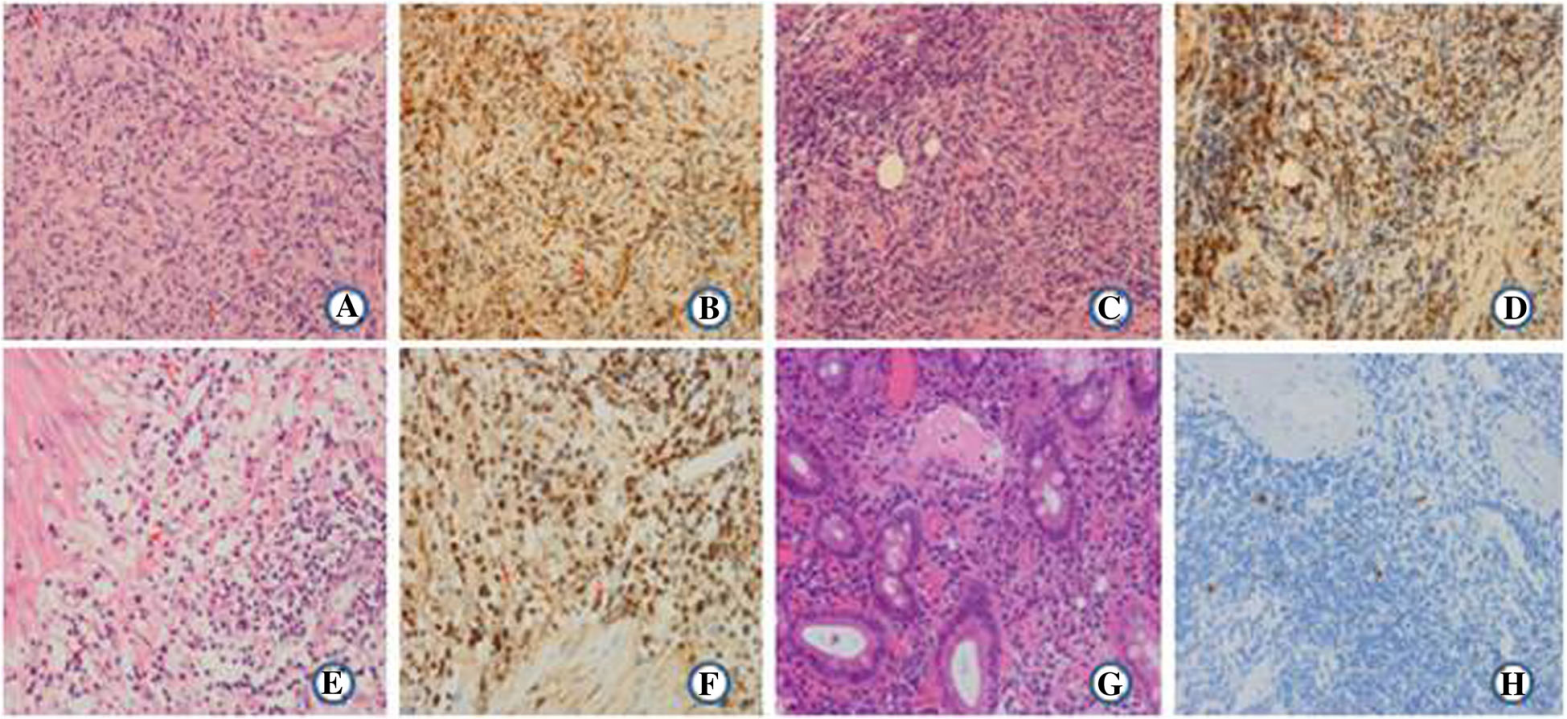
Pathologic findings. **a** Diaminobenzidine staining of liver specimens revealed focal necrosis of the liver surrounded by fibrous tissue. The focal necrosis area revealed infiltration by lymphocytes, plasma cells and eosinophils. Peripheral hepatocytes were partially silt. **b** Immunoglobulin G (IgG) immunohistochemical staining showing increased numbers of IgG-positive plasma cells in the liver lesions (30–60/ high power field [HPF], × 400). **c** Diaminobenzidine staining of pancreas tissues revealed focal areas of glandular atrophy accompanied by lymphocyte, plasma cell and eosinophil infiltration and fibrous tissue hyperplasia. **d** IgG immunohistochemical staining showing increased numbers of IgG-positive plasma cells in the pancreas (30–80/HPF, × 400). **e** Diaminobenzidine staining of gallbladder tissue revealed chronic cholecystitis, focal lymphoid hyperplasia, formation of lymph follicles and infiltration of the gallbladder wall by the lymphocytes, plasma cells and eosinophils. **f** IgG immunohistochemical staining showing increased numbers of IgG-positive plasma cells in the gallbladder (30–80/HPF, × 400). **g** Hematin and yepin staining of duodenum tissue revealed lymphocytic, plasmacytic and neutrophilic infiltration of the inner layer of the duodenal mucosa with congestion and dilatation of the blood vessels. **h** IgG immunohistochemical staining showing increased numbers of IgG-positive plasma cells in the duodenum (> 100 /HPF, × 400)

When investigating the recurrent bleeds, serum IgG4(0.035–1.500 g/L)and IgG(8~ 15.5 g/L)were found to be 29.200 g/L and 24.50 g/ L respectively and IgG4 disease was suspected (Table 1). The patient’s prior surgical pathology specimens were retrieved for IgG4 immunohistochemical examination. Immunohistochemical staining revealed increased IgG-positive and IgG4-positive plasma cells in the liver lesions (IgG4 positive cells 30–60/ high power field (HPF); Fig. 3b), pancreas (IgG4 positive cells 30–80/HPF; Fig. 3d) and gallbladder (IgG4 positive cells 30–80/HPF; Fig. 3f). Staining of the duodenum ulcer biopsy also showed more than 100 IgG4-positive cells per HPF (Fig. 3h). Based on these findings, a definitive diagnosis of IgG4-RD was made.

The patient was administered 40 mg/d prednisone intravenously for 7 days without any side effects followed by oral prednisolone 40 mg/d for 1 week. Laboratory tests indicated a reduction in the serum IgG4 levels 8 days after initiating prednisone but the liver function continued to be impaired (Table 1). As the patient improved symptomatically, he was discharged on a regime of prednisolone and mycophenolate mofetil.

Since discharge until the time of writing, during the 11 monthly visits since starting steroid therapy, laboratory tests indicate significant improvements in the patient’s serum IgG4 subtype, liver function and hemogram tests (Table 1).

## Discussion and conclusions

IgG4-RD was first described in patients with sclerosing cholangitis, known as autoimmune pancreatitis (AIP) type I. Subsequently, IgG4-RD was found to affect other organs [6]. At present IgG4-RD is believed to be a systemic disease and diagnostic criteria for IgG4-RD [8, 9] include: (1) single or multiple organs with diffuse or localized swelling, masses, nodules and/or hypertrophic lesions; (2) elevated serum IgG4 levels (≥135 mg/dL); and (3) histopathologic features that include marked lymphocytic and plasma cell infiltration and fibrosis, with IgG4-positive plasma cell infiltration (IgG4/IgG positive cell ratio of 40% and IgG4-positive plasma cells exceeding 10/HPF). Diagnosis of IgG4-related is confirmed when all of the following are fulfilled: (1), (2) and (3). Diagnosis is likely if criteria (1) + (3) are fulfilled, possible if (1) + (2) are fulfilled and unlikely if only (1) presents.

Notably, the biliary tract and pancreas are the organs most frequently affected by IgG4-RD [10]. Few studies have reported isolated cases of IgG4-RD in other digestive organs, such as in the esophagus, stomach, liver or duodenum [7].

A recent review of IgG4-RD cases reporting on gastrointestinal lesions showed that most patients present with multiple organ disease involvement, including the stomach, duodenum, liver, bile duct, esophagus, jejunum, lymph nodes and pancreas (Table 2). Six patients presented with obstructive jaundice as the main clinical manifestation [11–16] and others presented with varied clinical symptoms [17–28]. Gastrointestinal bleeding was not reported in any of the studies. Our patient had recurrent gastrointestinal bleeding due to a duodenal ulcer. The ulcer failed to heal with standard treatment. Serum IgG4 was elevated in most of the cases reviewed, similar to our case, though was normal in three reports [11, 13, 17] and was not reported in two cases [11, 18]. The diagnosis of IgG4-RD was based on a combination of features that include clinical parameters, serology, immunohistochemistry, imaging and histopathology. In the present case, the diagnosis of IgG4-RD was initially missed in the histological analysis of the liver and pancreatic biopsy and gallbladder samples. When IgG4-RD was suspected based on the clinical and laboratory findings, IgG4 immunostaining was undertaken and the IgG4-RD diagnosis was confirmed. In our literature review, there were eight patients treated through surgery, 10 patients by corticosteroids, two were given corticosteroids after surgery, and in two patient studies, the treatment course was not reported (Table 2). Our patient responded partially to steroid therapy and required other immunosuppressive agents for a complete and successful response.

**Table 2:** Summary of demographic, radiographic and clinical information from a review of 18 previously published cases of IgG4-Related gastrointestinal disorders

Study	Age, Gender	Clinical Symptoms	Laboratory	CNS Imaging (modality)	Biopsy Source	Organ Involvement	Treatment, Response
Fong et al., 2013 [11]	42, M	Obstructive jaundice, pruritus, pale stools, weight loss, tea-colored urine	Increased liver enzymes, IgG4 normal	Extrahepatic duct presented with a mural thickening (CT), stricture in the middle of bile duct with proximal biliary dilatation (ERCP)	Duodenal ampulla	Duodenal, liver	Marked
	79, M	Obstructive jaundice, weight loss	Not reported	Mild dilatation of the biliary tree (CT)	Not reported	Pancreas, biliary	Surgery
Rungsakulkij et al., 2017 [12]	56, M	Obstructive jaundice	Increased serum IgG4	Bile duct obstruction(CT); stricture of hepatic duct (ERCP)	Ampullary	Bile duct	Surgery
Cai et al., 2014 [13]	57, M	Jaundice, upper abdominal Discomfort	IgG4 serum normal	Mass observed in the hepatic duct (CT), hepatic duct and proximal bile duct dilatation (MRI)	Bile duct	Bile duct	Surgery
El Euch et al., 2017 [14]	70, M	Obstructive jaundice, anorexia, abdominal pain, \weight loss	Increased liver enzymes, and Serum IgG4	Diffuse pancreatic swelling and strictures of the main pancreatic duct (CT), thickened rim surrounding the pancreatic duct (MRI)	Not reported	Pancreatic	Marked
Miki et al., 2015 [15]	69, M	Jaundice, steatorrhea	Increased liver enzyme, and serum IgG4	Thickening of the bile duct wall, compressing the right portal vein (CT); bile duct lesions involving the left and right hepatic ducts (ERCP)	Bile duct	Bile duct	Surgery
Sivakumaran et al.., 2014 [16]	51, F	Jaundice, weight loss	Increased carbohydrate antigen 19–9	Intrahepatic duct dilatation and a hilar stricture (CT), a mass at the portal hepatitis (MRI)	Liver	liver	Surgery, Marked
Rodriguez et al., 2016 [17]	55, F	Abdominal pain, weight loss	IgG4 and other blood indices were normal	A mass in the pancreas(CT), hypermetabolic of the pancreas tail, bone marrow, and spleen, diffuse lymphadenopathy (PET)	Bone marrow	Pancreas	Marked
Kondo et al., 2016 [18]	78, M	Bilateral leg edema	Not reported	Sclerosing cholangitis (MRI)	Pleural	Pleura, bile duct, pericardium	Marked
Yang et al., 2015 [19]	60, M	Acid reflux	Increased serum IgG4	Multiple masses in the esophagus stomach, and liver (CT)	Esophagus, stomach	Esophagus, stomach and liver	Marked
Miyajima et al., 2017 [20]	50, M	Right upper pain, anorexia	Leucocytosis	Two liver masses (CT)	Liver	Liver	Surgery
Li et al., 2016 [21]	57, F	Pruritus	Increased liver enzymes and serum IgG4	Dilation of intrahepatic bile duct and lesions occupying the on head of pancreas, anastomotic stenosis (MRCP)	Biliary	Biliary	Surgery
Chen et al., 2016 [22]	58, M	Pruritus	Increased total bilirubin, direct bilirubin, lipase, and serum IgG4	Stricture of the distal bile duct and dilatation of the pancreatic duct (MRI); pancreatic duct with double duct sign (ERCP)	Not reported	Pancreas	Marked
Shimamura et al., 2015 [23]	74, M	Not reported	Increased serum IgG4	Low-density lesions, rim-like lesions in the bilateral kidneys (CT)	Gastric mass and Kidney	Stomach and kidney	Marked
Bulanov et al., 2016 [24]	62, F	Severe weakness and fatigue	Hemoglobin reduction, increased serum IgG4	Chronic ulcerative lesion, thickening of the stomach wall(CT)	Gastric mass and regional lymph nodes	Stomach, lymph nodes	Surgery
Takasumi et al., 2016 [25]	63, F	Not reported	thrombocytopenia, increased serum IgG4 and IgM	Enlargement of the submandibular glands, diffuse enlargement of the pancreas (CT)	Bone marrow, liver	Liver	Marked
Kim et al., 2016 [26]	61, M	Weakness, easy fatigability, weight loss	Increased liver enzymes and serum IgG4	Type IV hilar cholangiocarcinoma with periductal invasion into underlying hepatic parenchyma (MRI), multiple enlarged lymph nodes in left axillary (PET)		Intrahepatic bile duct	Marked
Matsunaga et al., 2014 [27]	72, M	Not reported	Increased hepatobiliary enzymes, serum IgG, and tumor markers	Enhancement in the pancreatic head, stenosis in the bile duct head and dilatation (CT), diffuse pancreatic ductal stenosis (MRI)	Duodenal papilla	Duodenum	Not reported
Van et al., 2017 [28]	26, M	Lymphadenopathy, splenomegaly	Increased IgG4, reduced T lymphocyte	Not reported	Pancreas	Pancreas	Not reported

*CT* computed tomography, *ERCP* endoscopic retrograde cholangiography, *MRI* magnetic resonance imaging, *PET* positron emission tomography

IgG4-RD can affect multiple gastrointestinal organs simultaneously or over different time periods. The clinical manifestations can mimic malignancy or other benign diseases such as portal hypertension, acid peptic disease. A high index of suspicion is required to make an accurate diagnosis and avoid unnecessary surgical interventions.

## Abbreviations

ALT: Alanine aminotransferase
AST: Aspartate aminotransferase; ALB: albumin
CRP: C-reactive protein
CT: Computed tomography
DBIL: Direct bilirubin
ERCP: Endoscopic retrograde cholangiography;
HGB: Hemoglobin
IgG: Immunoglobulin G
IgG4: Immunoglobulin G4 subtype
IgG4-RD: IgG4-related disease
MRI: Magnetic resonance imaging; PET: positron emission tomography
PLT: Platelet
TBIL: Total bilirubin
